# Temporal Video Segmentation Approach for Peruvian Sign Language

**DOI:** 10.3390/s25175217

**Published:** 2025-08-22

**Authors:** Summy Farfan, Juan J. Choquehuanca-Zevallos, Ana Aguilera, Irvin Dongo, Yudith Cardinale

**Affiliations:** 1 Electrical and Electronics Engineering Department, Universidad Católica San Pablo, Arequipa 04001, Peru; jchoquehuancaz@ucsp.edu.pe (J.J.C.-Z.); ifdongo@ucsp.edu.pe (I.D.); 2 Department of Signal Theory and Communications, Universidad Carlos III de Madrid, 28911 Leganes, Spain; 3 Escuela de Ingeniería Informática, Facultad de Ingeniería, Universidad de Valparaíso, Valparaíso 2340000, Chile; ana.aguilera@uv.cl; 4 MEDING Interdisciplinary Center, Facultad de Ingeniería, Universidad de Valparaíso, Valparaíso 2340000, Chile; 5 ESTIA Institute of Technology, University of Bordeaux, 64210 Bidart, France; 6 Centro de Estudios en Ciencia de Datos e Inteligencia Artificial (ESenCIA), Universidad Internacional de Valencia, 46002 València, Spain; ycardinale@universidadviu.com

**Keywords:** temporal segmentation, Peruvian Sign Language, diffusion network, deep learning

## Abstract

Continuous Sign Language Recognition is a task that involves the recognition of complete sequences of Sign Language videos and translating them into spoken or written language. This task is particularly challenging due to the complex temporal structure and variability in signing styles. In this context, temporal video segmentation emerges to distinguish individual signs from transitions in continuous video streams. Despite its importance, temporal segmentation for Sign Language has not yet fully benefited from recent advancements in Machine Learning. Many existing approaches still rely on outdated architecture. This study aims to learn the characteristics that distinguish signs from transitions, which are fundamental elements of Sign Language. To this end, we adapt two current temporal segmentation models, DiffAct and MS-TCN, and apply them to our own precisely annotated datasets for Peruvian Sign Language. We explore three training strategies—baseline, data augmentation, and multi-dataset. Results suggest that they can enhance the scores for both models but at the cost of increased variability across splits. Notably, the diffusion-based model showcased its ability to generalize to unseen sequences through higher scores for sign and transition identification on the test set, reaching a median value of 71.89% for mF1S and 72.68% for mF1B.

## 1. Introduction

Disability inclusion has become a global priority, aiming to create fairer societies where all individuals can fully participate. This is critical for the 1.3 billion people worldwide who experience some form of disability [1]. Despite advancements in terms of policy, barriers still persist, including poverty resulting from limited access to education and social exclusion due to prejudice and discrimination. In Peru, the 2017 national census reports that 10.3% of the population (3.2 million individuals) deals with disability, with the majority being women [2]. Within this group, 60.4% of the working-age disabled population is economically inactive, and many do not have access to insurance coverage. Educational disparities aggravate the issue: 13.4% of individuals with disabilities have no formal education and 16.8% remain illiterate, with only 0.5% accessing specialized education.

In 2017, deafness was the third most common disability in Peru, affecting 7.6% of the disabled population (243,486 individuals) [2]. Although Peruvian Sign Language was officially recognized as a national language back in 2010 by Law No. 29535, it is not considered a true language by many linguistic groups, so it is not widely accepted or utilized [3]. As a consequence, many deaf children are pressured to learn oral language and lip-reading, which are ineffective for those unable to associate words with sounds. This lack of acceptance, coupled with limited exposure to Peruvian Sign Language during critical developmental years, often results in language deprivation, which can negatively impact cognitive development, mental health, and overall well-being [4].

Recently, many efforts have been focused on creating digital services to support education and social inclusion of people with hearing impairments [5]. In particular, automatic and Continuous Sign Language Recognition has attracted the interest of the research community [6]. To do so, Machine Learning has been increasingly applied to Sign Language processing [7,8,9,10,11,12]. At first, studies focused on Isolated Sign Language Recognition (ISLR), a task that classifies individual signs from pre-cropped video segments and for which various methods have been proposed [13,14,15]. More recently, the focus has moved towards Continuous Sign Language Recognition, which aims to recognize entire sentences and provide full translations [8,10,16,17]. In this context, temporal video segmentation emerges as a way to distinguish individual signs from transitions in continuous video streams. As stated by Koprinska and Carrato [18], the main objective of Continuous Sign Language Recognition is to divide a video stream into meaningful and manageable segments, making it the first step toward the automatic annotation of digital video sequences.

In this regard, by learning differences between signs and transitions, temporal segmentation models can improve the flexibility and accuracy of Sign Language recognition systems [19,20]. Moreover, this characteristic can speed up the labeling process, as spotting the temporal limits of signs is the most complex and time-consuming task.

Despite its importance, temporal segmentation models for Sign Language have not yet benefited from recent developments in Machine Learning (many existing approaches still rely on foundational models such as the work shown in Renz et al. [21]).

While temporal segmentation has been extensively studied in the context of general actions, adapting these models to Sign Language presents unique challenges. Unlike actions, which benefit from rich contextual and environmental cues such as full-body motion and object interaction, Sign Language conveys meaning primarily through facial expressions and hand movements. This narrower visual scope limits the available information for segmentation, requiring more precise modeling of subtle gestures and transitions [22].

Building upon the outlined challenges, this work is the first to apply temporal segmentation specifically to Peruvian Sign Language. It addresses a significant gap in the literature and seeks to inspire related future research through its key contributions:

- Precise temporal annotation of three Peruvian Sign Language datasets. We annotated 546 videos across three distinct datasets, with a total duration of 57.64 min of continuous video stream. Special emphasis was placed on accurately identifying the temporal boundaries between signs and transitions, addressing a major gap in existing Peruvian Sign Language datasets. These high-quality annotations provide a valuable benchmark for training and evaluating temporal segmentation models, supporting future research in the field.
- Development of a Binary Temporal Segmentation Framework for Peruvian Sign Language datasets. A customized Deep Learning pipeline was designed to detect transitions and signs within continuous video streams, addressing the challenges posed by limited annotated data.
- This work marks the first known application and adaptation of two state-of-the-art models—originally developed for different domains and tasks—to the context of Peruvian Sign Language. By retraining these models on the newly annotated Peruvian Sign Language datasets, we enable a comparative evaluation of their performance and capacity to generalize in a low-resource Sign Language setting.

In summary, this paper aims to advance the field of Peruvian Sign Language temporal segmentation by developing a new dataset, adapting state-of-the-art models from temporal action segmentation, and assessing their effectiveness.

This paper is organized as follows. A summary of recent research is given in Section 2, with an emphasis on action temporal segmentation and Sign Language processing. Section 3 describes our methodology, detailing the dataset creation, model adaptations, and experimental setup. The results on the test and validation sets are analyzed both quantitatively and qualitatively in Section 4. We conclude and propose future perspectives of our work in Section 5.

## 2. Related Work

In this section, we describe both early approaches based on thresholding and more recent Deep Learning methods for temporal sign segmentation. Additionally, we review temporal segmentation techniques applied in other domains—such as actions and Sign Language comparison—to provide a broader context for methodological advances relevant to our study.

### 2.1. Threshold-Based Methods

Early work on temporal segmentation was addressed using thresholds. Mocialov et al. [23] propose the use of static thresholds to differentiate small, medium, and large movements by calculating hand centroid trajectories, assuming that acceleration is lower during transitions, making it possible to differentiate transitions from signs. Similarly, Choudhury et al. [24] classify small movements as transitions, while Mocialov et al. [23] also include medium movements in the same category.

However, centroids consider only global hand movement, excluding the motion of fingers, which is crucial for accurately capturing sign configuration required to differentiate signs. To address this limitation, Nayan et al. [25] propose adaptive thresholds, which calculate optical flow to estimate the velocity of hands and fingers, and then two-norm values of the magnitude matrix are used to differentiate signs from transitions. This strategy is applied to finger-spelling in Indian Sign Language, based on the assumption that sign frames are quasi-stationary, which is true only in this specific scenario of spelling. Another strategy is proposed by Farag and Brock [26], who base their proposal on 41 three-dimensional articulation positions as data input. Then, geometric descriptors are used to model the spatial relationship and translation movement, and temporal information is added through sliding windows. A balanced binary random forest, enhanced with bootstrapping, is employed to classify sequences of frames as either a sign or a non-sign.

Although these studies demonstrate the utility of threshold-based methods, they also highlight their limitations. These methods tend to oversimplify the movements in Sign Language or make assumptions that fail to capture its full complexity.

### 2.2. Deep Learning Methods

Recent advances have focused on Deep Learning techniques capable of modeling complex temporal dependencies. Yang et al. [27] identify that the reduction in temporal resolution, a common issue in sequential data processing, is associated with the use of pooling layers, as strides are often greater than 1. Therefore, their study suggests modifications to the architecture of Shou et al. [28] by replacing convolutional filters with Temporal Preservation Convolution (TPC)—dilated convolutions—along the temporal dimension while maintaining the same parameters for the spatial dimension.

Pérez et al. [22] adapted ASFormer, a Transformer-based architecture originally proposed by Yi et al. [29] for action segmentation, to the task of Sign Language segmentation using five-fold cross-validation. ASFormer models temporal sequences through encoder blocks that combine self-attention mechanisms with dilated temporal convolutions, allowing the capture of complex temporal patterns within predefined windows. The decoder comprises multiple cross-attention blocks that progressively refine predictions by integrating external information, thereby reducing error accumulation.

In their adaptation, Pérez et al. [22] reported lower performance compared to action segmentation, primarily due to the removal of positional encoding and the intrinsic differences between the two tasks. While action sequences typically involve slower, goal-oriented movements and interactions with the environment, Sign Language is characterized by rapid manual articulations and non-manual cues such as facial expressions. These features introduce additional complexity in temporal modeling, which poses challenges for effective segmentation.

Renz et al. [21] retrained the MS-TCN architecture, originally proposed by Farha and Gall [30], on the BSL-Corpus and PHOENIX14 datasets, achieving higher results over ASFormer. The MS-TCN model consists of four stages with ten dilated convolutional layers each, where the dilation factor doubles at every layer using 64 filters of size 3. This structure enables the model to capture increasingly long temporal dependencies without resorting to pooling or fully connected layers, thereby preserving temporal resolution and avoiding parameter inflation. Importantly, each stage outputs only per-frame class probabilities, as incorporating additional features was found to degrade performance. To enhance temporal consistency, the model integrates two loss functions during training: cross-entropy loss for frame-wise classification and a smoothing loss to penalize abrupt transitions. However, the qualitative analysis revealed over-segmentation issues, especially for short signs and fingerspelling, as each stage introduced incremental segmentation errors that negatively impacted the F1-score.

Temporal segmentation has also been widely explored in the context of action recognition, which Ding et al. [31] define as the task of segmenting untrimmed videos and assigning each segment a label from a predefined set of actions. Bahrami et al. [32] propose LTContext, a model that combines local windowed attention with long-term sparse attention to effectively capture both short-term and long-range dependencies. The architecture employs blocks composed of temporal convolutions followed by attention mechanisms, demonstrating the importance of balancing local and global context, particularly in lengthy and complex action sequences.

Liu et al. [33] introduce DiffAct, a generative model based on denoising diffusion processes. It consists of an encoder that progressively adds noise to the video sequence and a decoder trained to reconstruct the original sequence, following a generative refinement paradigm. In contrast, Wen et al. [34] develop a real-time segmentation framework that operates in a single stage. Their model incorporates a temporal convolutional network with a memory cache, a spatial–temporal feature extractor, and multimodal inputs by integrating language prompts with image features. This approach achieves competitive accuracy while maintaining computational efficiency and reducing over-segmentation.

While models used in temporal action segmentation have leveraged recent advancements in Machine Learning, those applied to temporal segmentation for Sign Language often rely on outdated architectures. These approaches could benefit significantly from state-of-the-art techniques that are specifically designed to capture long-range temporal dependencies in video data.

However, it is important to note that temporal segmentation is not the only approach to transitioning from isolated to Continuous Sign Language Recognition. Several studies have addressed this challenge using alternative strategies that do not rely on explicit segmentation [8,35,36].

Huang et al. [35] argue that temporal segmentation is a source of errors that propagate through subsequent stages of a recognition model. Therefore, they propose a Hierarchical Attention Network with Latent Space (LS-HAN). For feature extraction, two-stream 3D Convolutional Neural Network (CNN) is employed, where one stream captures hand location and motion while the other focuses on local characteristics of local hand characteristics, LS maps video representations and annotations into a shared space, and the HAN predicts the sentences word by word. To achieve this, the authors use two large-scale datasets, the Modern Chinese Sign Language dataset and the PHOENIX14 dataset, both of which are limited to a given number of signs.

A recent model proposed by Feng et al. [8] on Continuous Sign Language Recognition (CSLR), focuses not only on the correct classification of the sentences but also on the semantic coherence of the output. The proposed model is composed of four main modules: a dynamic feature module, a sequence module, a cross-modal contrastive learning module, and a classifier. The dynamic feature module employs dynamic trajectory capture and a key enhancer in order to highlight motion trajectories and relevant Sign Language elements. Next, temporal convolution is combined with Bi-LSTM to model temporal dependencies. The third module aligns visual and textual representations at the gloss level. For the classification module, ResNet34, pre-trained on ImageNet, is used asthe backbone.

A main drawback is that CSLR requires access to large-scale annotated datasets to achieve high performance. However, such datasets are scarce and costly to produce, which is why most existing studies rely on a limited number of publicly available benchmarks. While effective within their constrained settings, these approaches face significant limitations when applied to other Sign Languages. In particular, they often depend on a fixed vocabulary and require multiple repetitions per word or sentence, making them less suitable for low-resource languages or real-world deployment where such data is not available.

A summary of the related works discussed in this section is provided in Table 1. Notably, the majority of these studies are based on the PHOENIX14 dataset, which offers multiple sentence-level annotations and serves as a widely accepted benchmark in the CSLR field.

## 3. Temporal Segmentation for Peruvian Sign Language: Our Proposal

**Figure 1:**
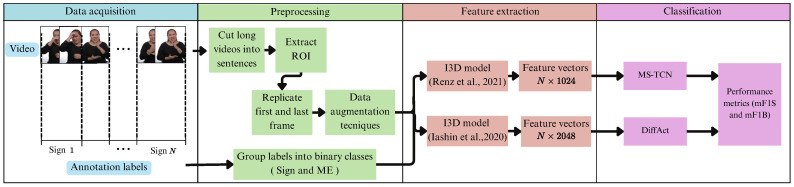
The overall pipeline of the proposed framework. Renz et al. [21], Iashin et al. [37].

As shown in Figure 1, our proposed framework for temporal segmentation for Peruvian Sign Language consists of a four-step approach: data collection, preprocessing, feature extraction, and classification. Our approach involves the use and comparison of two Machine Learning models: DiffAct and MS-TCN.

DiffAct is a diffusion-based model with an encoder–decoder architecture, originally developed for action temporal segmentation. It models the data distribution through iterative denoising and has demonstrated robust performance across different datasets, outperforming transformer-based models [33]. These results motivated its selection to investigate whether its architectural advances can improve temporal segmentation in Sign Language. Therefore, in this work, we adapt DiffAct for the first time to Peruvian Sign Language. MS-TCN is a multistage model, related to temporal segmentation for Sign Language, which has demonstrated state-of-the-art performance, specifically on British and German datasets. It was selected as the baseline in our work, since it is the first and only model formally adapted for temporal segmentation in Sign Language, offering a strong reference to evaluate cross-language transferability. We excluded other potentially beneficial models due to practical constraints, as many lack reproducible implementations or accessible pretrained weights, making fair and controlled comparisons unfeasible. In contrast, MS-TCN and DiffAct provide publicly available, well-documented codebases and pretrained models, enabling reliable evaluation on our dataset.

The following sections describe in detail each step of our proposed approach.

### 3.1. Data Acquisition

We evaluated several available Peruvian Sign Language datasets, and we realized that they do not provide accurate boundaries of individual signs within continuous video streams, limiting their applicability to this research. In the following, we describe the limitations of the Peruvian datasets evaluated.

**Figure 2:**
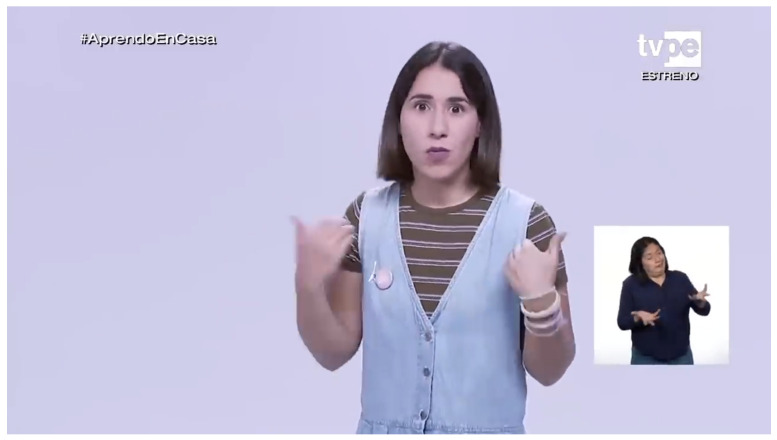
Example frame from AEC PUCP dataset [38].

The AEC PUCP dataset [38] was developed by the Pontificia Universidad Católica del Perú (PUCP) and created from videos of the educational program *Aprendo en casa* (learning at home), a national TV program that started its transmission during the pandemic, around 2020, which allowed children with access to TV or internet to keep studying the basic subjects, as online teaching was not possible at the time. The program’s topics are very varied and depend on children’s ages. The videos consist of a teacher explaining a certain topic, while an interpreter, located at the lower corner, is translating it into Peruvian Sign Language. An example frame from the dataset is shown in Figure 2. The AEC PUCP is the largest Peruvian dataset; it contains two videos partially annotated by three volunteers with intermediate knowledge of Peruvian Sign Language. Two levels of annotations are used: word and sentence. However, when reviewing the dataset, the boundaries of words were imprecise, making the dataset not useful for temporal segmentation.

**Figure 3:**
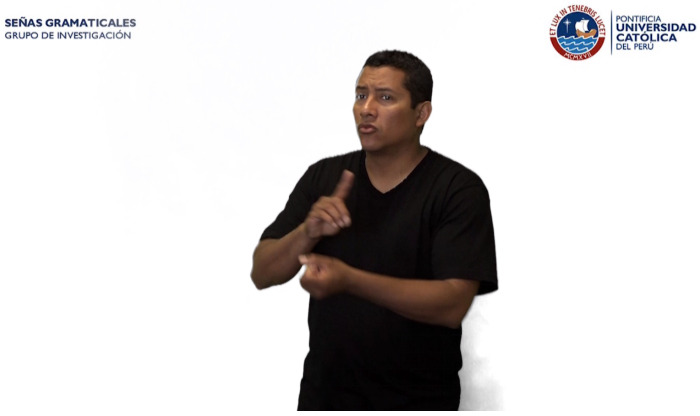
Example frame from PUCP-305 dataset [39].

PUCP-305 [39] is a very varied dataset, as five interpreters participated in the video filming process. Each video features a signer standing in front of the camera, dressed in black, as illustrated in Figure 3, signing predefined sentences, with only one repetition per sentence. However, their participation was not even, resulting in an imbalanced dataset. Also, the boundaries of words were poorly annotated.

It is important to point out that AEC PUCP and PUCP-305 were annotated using ELAN, a software tool that allows assigning labels to specific time segments.

Lastly, LSP10 dataset [40] contains videos of 25 people standing in front of varied backgrounds, facing a Kinect sensor v1 and wearing different types of clothing. The participants perform 10 short sentences, used on a daily basis, with each sentence repeated 60 times. Nevertheless, there is no level of annotation, as the video names are used as the only way of labeling.

A summary of the characteristics of the state-of-the-art Peruvian datasets are presented in Table 2, highlighting their limitations for training Machine Learning models in temporal segmentation. To bridge this gap, we introduce three refined datasets, manejar_conflictos (manage_conflicts), ira_alegria_RE (anger_joy), and PUCP-305_RE, where “RE” stands for refined. The annotations for the three datasets were newly created in this work, but the video content was obtained from existing sources. Frame-level annotation was conducted to precisely mark the boundaries between signs and transitions. The following section provides a detailed description of these datasets.

#### 3.1.1. Datasets

The manejar_conflictos and ira_alegria_RE datasets originate from two 28-second-long videos produced by *the Aprendo en casa* TV program, which are publicly available on YouTube. The PUCP-305_RE dataset was curated from the original PUCP-305 dataset [39] and includes a selection of 174 videos. This selection was made by a Peruvian Sign Language interpreter, who identified the most relevant and frequently used sentences to construct a more focused and representative dataset. Although we did not record the videos ourselves, they were originally produced under controlled conditions, ensuring consistency in their characteristics:

- Interpreters wear dark clothing.
- The background is uniformly white.
- Videos were recorded at an approximate frame rate of 30 fps.
- The camera is positioned directly in front of the signer, capturing their faces and keeping the signing space within the frame.

The annotation process was conducted manually by a certified Peruvian Sign Language interpreter using ELAN software, ensuring high-quality and consistent labels throughout the datasets. Each sentence in the datasets is unique, with no repetitions. A detailed summary of the dataset characteristics is provided in Table 3.

#### 3.1.2. Annotation Process

The annotation process involves assigning a specific label to a recorded time span, where a sign has been spotted. We follow the guidelines proposed by Cormier and Fenlon [41], where the end of a sign is marked when the hands begin to move away from the previous sign, while we consider the start of a sign when the hands reach the minimal position and configuration necessary to perform the sign.

Following this approach, every sign is labeled with its corresponding word in Spanish. Based on the recommendations of Bejarano et al. [42], the words are written in the present tense and in their masculine form. Additionally, we ensure that the same sign is represented by a single word. It is important to point out that the annotation process is a very slow and costly task to perform, as every minute of the video takes 1 h to annotate.

Beyond the two primary categories—*sign* and *transition*—other categories such as *rest*, *gestural signs*, *fillers*, and *NN* (incorrectly or incompletely performed signs) are also identified in the datasets, which naturally occur in the flow of a conversation. These categories are detailed in Table 4. *Fillers* and *NN* often lack clear semantic content, are challenging even for human annotators, and can negatively affect the model’s ability to learn meaningful temporal boundaries.

Identifying these categories in continuous video streams is crucial, as ignoring them could hinder sign recognition. However, using a multiclass approach is not possible, because they occur less frequently than the two main classes, as stated in Table 5. Thus, we maintained a binary classification approach by changing labels. To this end, *fillers*, *NN*, and *gestural signs* were grouped under the *sign* label due to their similarity to this class, while *rest* was labeled as *transition*. Additionally, Table 5 shows that transitions are inherently fewer than sign frames, resulting in a class imbalance at a ratio of approximately 1:2. To mitigate this skewed distribution, we adopted a cost-sensitive learning strategy by leveraging the pos_weight parameter in the binary cross-entropy loss. As recommended in the PyTorch documentation, this weight was computed as the ratio between the majority (*sign*) and minority (*transition*) class frequencies, effectively rebalancing the loss function to penalize misclassifications of the shorter transition segments more heavily. This adjustment enhances gradient contributions for the underrepresented transition class.

**Figure 4:**
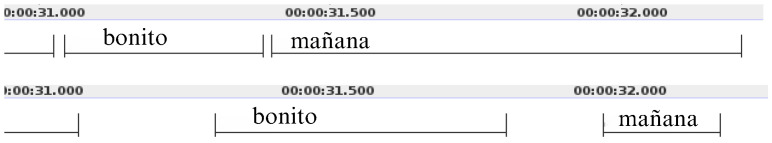
Comparison between the original annotation of the video ira_alegria in the dataset AEC PUCP (upper image) and the refined annotation for the new dataset ira_alegria_RE (lower image).

As a demonstration, Figure 4 provides a comparison between the annotations of the dataset AEC PUCP for the ira_alegria video with those in the dataset ira_alegria_RE, highlighting key differences that significantly impact the quality of the data used to train the Machine Learning model, because, depending on the precision of annotation, we either include or exclude a given number of frames, which affects the delimitation between closely related classes.

### 3.2. Preprocessing

Firstly, the videos were cropped to isolate the Region of Interest (ROI) surrounding the Peruvian Sign Language interpreter, ensuring the full signing space was included. To ensure compatibility with the feature extraction models, the video frames were resized to a fixed resolution of 220×220 pixels before being fed into the models. Moreover, the videos were temporally trimmed to last between 4 and 8 s, encompassing 7 to 14 signs per video. As a result, we obtained 171 videos for the dataset manejar_conflictos, 201 for ira_alegria_RE, and 174 sentences were already clipped for PUCP-305_RE.

#### Data Augmentation

The goal of data augmentation is to enhance the quality, volume, and diversity of the training data, as collecting sufficient data for real-world applications is difficult and costly [43]. To address this, data augmentation techniques were applied to the datasets by introducing random variations in rotation, zoom, and translation. Their specific variation ranges are detailed in Table 6.

These techniques fall under the category of affine transformations. Affine transformations are geometric modifications that alter the position of pixels without changing their values [43]. They are considered traditional data augmentation techniques and are widely used to make training data more representative of real-world variations. Despite their simplicity, these methods have proven effective in improving the performance of Machine Learning models, so that they are usually applied before more advanced techniques.

The selected transformations expanded the dataset while preserving the linguistic and gestural integrity of the signs, making them suitable for training without introducing unrealistic variations. Other common augmentation techniques, such as frame sampling or inserting artificial intermediate frames, were avoided because they could alter the natural sequence of movements, leading to unrealistic transitions that do not accurately represent real-world Sign Language patterns. By focusing on spatial augmentations rather than temporal modifications, the approach ensured that the model learned from data that remained true to actual signing behavior while still benefiting from additional variability.

An ablation study was conducted to assess the impact of these augmentation techniques on segmentation performance. The experiments conducted consist in assessing the results obtained in the validation dataset when adding augmented versions of the data to the training phase. To identify the most effective one, all possible combinations of rotation, zoom, and translation transformations were evaluated. Notably, the transformations were carried out by using openCV functions.

### 3.3. Feature Extraction

Since videos cannot be processed directly, feature extractors were required to obtain the most representative information, reducing the amount of data the Machine Learning models need to process. In this study, we employed the I3D feature extractors proposed by Renz et al. [21] and Iashin et al. [37], leveraging the prior knowledge embedded in these architectures.

**Figure 5:**
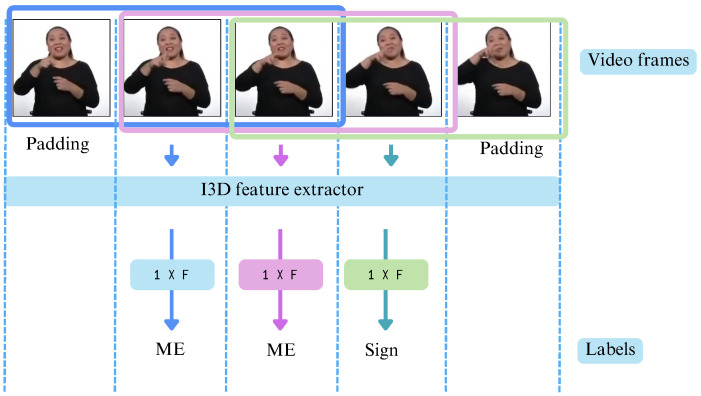
Illustration of the sliding window mechanism used for feature extraction.

These feature extractors’ selection depends on the I3D input shape required by the models used in this study, MS-TCN and DiffAct. The key difference between them lies in the shape of their output feature vectors:

- The extractor of Renz et al. [21] produces a feature vector of size N×1024,
- The extractor of Iashin et al. [37] generates a feature vector of size N×2048,

where *N* represents the number of temporal windows in the video, as the feature extractors compute RGB and optical flow features using sliding windows, with each resulting vector being assigned to the middle frame. This process is illustrated in Figure 5, where a simplified example is shown using a window size of w=3 and a stride of 1.

To ensure that predictions align with the ground truth labels for effective model comparison, videos were padded by repeating the first and last frames. The number of repetitions is determined by the window size *w*, which varies depending on the feature extractor. Specifically, Renz et al. [21] use a fixed window size of w=16, as it is hardcoded in the implementation, while Iashin et al. [37] employ the default setting of w=21. In both models, the stride is set to 1.

### 3.4. Classification

As we mentioned, MS-TCN [21] is the most recent work in temporal segmentation for Sign Language and serves as the state-of-the-art reference model for our study. Given its strong performance and its origins as an adaptation of an action segmentation model, we adopt a similar methodology by leveraging transfer learning. In the case of DiffAct, knowledge acquired from action segmentation is repurposed to improve temporal segmentation in Peruvian Sign Language, allowing for an assessment of both its effectiveness and limitations in this specific context.

Instead of adopting a Continuous Sign Language Recognition approach, this study focuses on temporal segmentation due to the characteristics of the Peruvian Sign Language dataset. Unlike large-scale international datasets, such as PHOENIX14T [44] or BSL-Corpus [45], which are for German and British Sign Language, respectively, the datasets used in this study are significantly smaller, limiting the feasibility of training deep models from scratch. Additionally, the datasets contain unique sentences, meaning that the sequence of signs is never repeated, making the segmentation task inherently more challenging.

Furthermore, this study addresses the common challenge of limited annotated data, since the goal is to assist interpreters in accelerating the annotation process. The models are designed to generalize from the available data, predicting the location of signs and transitions even when encountering unseen sign sequences. Although the predictions may not be perfectly accurate, they can significantly reduce the time required for manual annotation, making the process more efficient.

#### 3.4.1. Model A: MS-TCN

Renz et al. [21] state that the MS-TCN model was retrained on the BSLCorpus and PHOENIX14 datasets. Originally designed for temporal action segmentation, MS-TCN employs a multi-stage architecture in which initial predictions are iteratively refined through successive stages. This recursive approach provides higher-level layers with extended contextual information, enabling the capture of dependencies between actions.

Each stage outputs per-frame class probabilities without incorporating additional features, as including extra information was found to degrade performance. Furthermore, the model omits pooling and fully connected layers to preserve temporal resolution and limit the number of parameters. Instead, every stage consists solely of temporal convolution layers, with an increased receptive field achieved through 1D dilated convolutions. Also, there is a residual connection between the input and output of each dilated convolution layers.

During training, a combination of two loss functions is used:

- Cross-entropy loss ($L_{cls}$) is applied for classification.
- Smoothing loss ($L_{T-MSE}$)—defined as the truncated mean squared error over the logarithmic per-frame probabilities—is employed to mitigate the problem of over-segmentation. The contribution of the smoothing loss is regulated by the λ factor.

**Figure 6:**
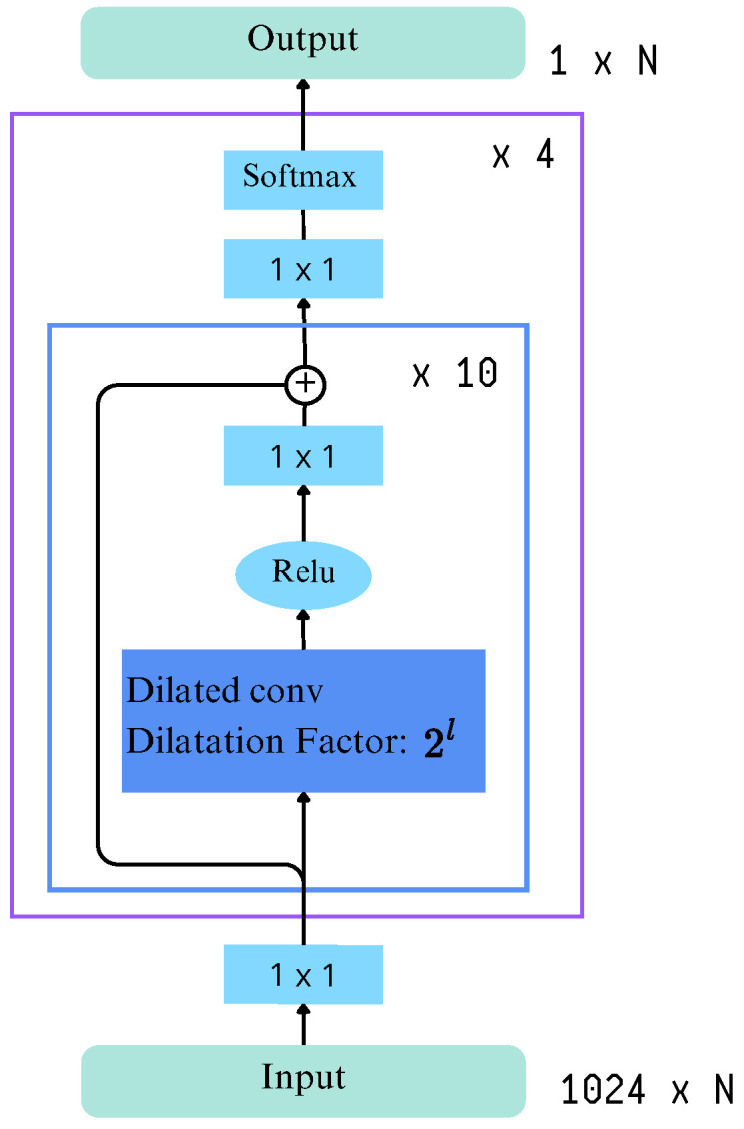
Architecture of MS-TCN.

MS-TCN [21] adopts the architecture and hyperparameters used by Farha and Gall [30], that is, an architecture composed of four stages, each one containing 10 dilated convolution layers. The dilation factor doubles with each subsequent layer, and each layer uses 64 filters of size 3. Figure 6 shows the overall architecture with its four stages of dilated temporal convolutions and residual connections. These connections progressively refine predictions at each stage. The input represents feature embeddings from video frames, while the output corresponds to frame-wise class probabilities.

#### 3.4.2. Model B: DiffAct

DiffAct, proposed by Liu et al. [33], is a diffusion-based generative model, structured as an encoder–decoder architecture and designed for temporal action segmentation. Unlike conventional discriminative models that perform frame-wise classification, it incorporates a denoising diffusion process, enabling the model to learn the underlying distribution of action sequences. This approach enhances temporal consistency and boundary alignment, addressing key challenges in action segmentation.

**Figure 7:**
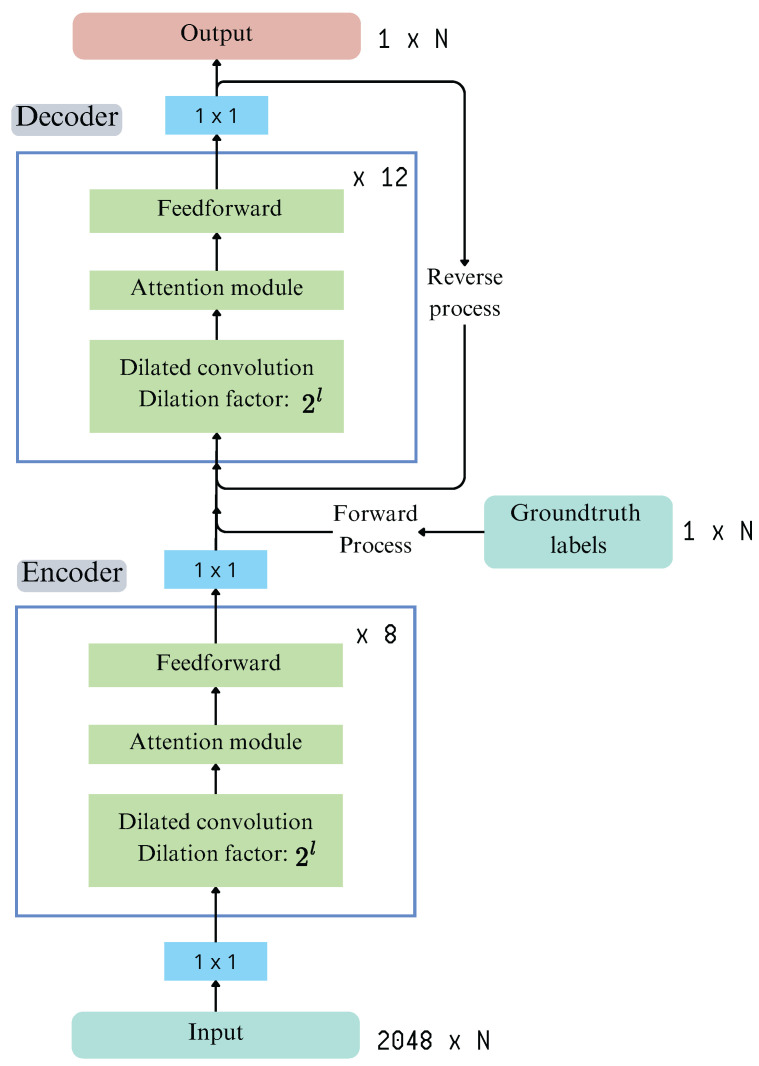
Overview of the DiffAct model presented in Liu et al. [33].

Moreover, the model follows a two-step generative framework, as pictured in Figure 7. The forward process gradually corrupts input features by adding Gaussian noise at each step with a predefined variance, while the reverse process iteratively removes noise, reconstructing the original action sequence. This iterative refinement helps the model to generate more coherent predictions while reducing frame-wise inconsistencies.

The encoder processes I3D features (F=2048), extracted from the input video, and generates a temporally enriched representation. It consists of 12 convolutional blocks, each containing 256 feature maps and a kernel size of 5. To enhance long-term dependencies, dilated convolutions are incorporated, effectively expanding the model’s receptive field. A key design choice is the multi-scale feature extraction, where the outputs of blocks 7, 8, and 9 are concatenated before being passed to the decoder. This approach retains information at multiple temporal resolutions, improving segmentation accuracy.

Before entering the decoder, the feature representations undergo masking and noise injection. Specifically, Gaussian noise is added to simulate a diffusion process, which helps the model learn to denoise and refine predictions. Additionally, random feature masking is applied, forcing the model to reconstruct missing information. This strategy improves generalization by making the model more robust to variations in input data.

The decoder follows a structure similar to the encoder but is optimized for sequence reconstruction. It consists of 8 convolutional blocks, each with 128 feature maps and a kernel size of 7, which improves smoothing and feature refinement. Additionally, self-attention layers are incorporated to enhance the model’s ability to focus on relevant temporal dependencies, ensuring that predictions align with the true structure of the action sequence. The decoder removes noise iteratively, restoring the original sequence from a noise-corrupted input, which results in more coherent and temporally consistent action segmentation.

On the other hand, DiffAct optimizes a weighted multi-term loss function, ensuring accurate classification, smooth transitions, and well-defined boundaries. The total loss is defined as in Equation (1), where

- $L^{ce}$ (Cross-entropy loss) supervises multi-class classification, ensuring accurate action predictions.
- $L^{bd}$ (Binary cross-entropy loss) detects class transitions, assigning 1 to boundary frames and 0 otherwise.
- $L^{smo}$ (Mean squared error loss) enforces temporal smoothness by minimizing variations between neighboring frames.

(1) $$L^{sum}=\alpha \times L^{ce}+\beta \times L^{bd}+\gamma \times L^{smo}$$

Initial weight settings are α=0.5, β=0.1, and γ=0.1.

#### 3.4.3. Performance Metrics

In order to evaluate the performance of the resulting models, we use the performance metrics proposed by Renz et al. [21]. These metrics assess how accurately the models identify contiguous segments of video frames corresponding to either *signs* or *transitions*, within a continuous sequence, as detailed below:

- mF1B (mean F1 Boundary): This metric evaluates the accuracy of predicted boundaries in action segmentation. A boundary prediction is considered correct if its midpoint-to-true-boundary distance is within a specified threshold (from 1 to 4 frames). The final mF1B score is obtained by averaging the F1B scores computed for each threshold in this range, providing a comprehensive measure of boundary detection accuracy.
- mF1S (mean F1 Sign): This metric measures the quality of sign segments in temporal action segmentation. A predicted segment is considered correct if its Intersection over Union (IoU) with the ground truth exceeds a threshold (ranging from 0.45 to 0.75). The final mF1S score is calculated as the mean F1-Score across all thresholds, ensuring a robust evaluation of both temporal alignment and segment completeness.

The practical implications of these metrics extend beyond simple per-frame classification as they reflect the model’s capability to accurately capture complete temporal segments rather than merely predicting isolated frames.

## 4. Experiments and Analysis

This section presents the experimental framework used to compare the MS-TCN and DiffAct models, detailing the implementation setup, training procedures, and evaluation metrics. We provide a comprehensive analysis of the results obtained, including ablation studies that explore the contribution of individual components and design choices within the model. Through these experiments, we aim to validate the effectiveness of our approach and gain deeper insights into the factors influencing performance.

**Datasets**. This study utilizes three datasets (depicted in Table 3): manejar_conflictos, ira_alegria_RE, and PUCP-305_RE. For the initial training phase, only the manejar_ conflictos dataset was used, as it provides the most suitable data for model training in terms of clarity and class separability. Specifically, this dataset contains fewer instances of ambiguous or non-discriminative categories—such as *filler* and *NN* segments. The ira_alegria_RE dataset, which includes a high proportion of these categories, was initially excluded for this reason, but was later incorporated to explore its potential in enhancing model robustness. The PUCP-305_RE dataset was omitted from training due to an imbalanced distribution of videos among signers, which could lead to underrepresentation and hinder generalization.

For both validation and testing, only the manejar_conflictos dataset was employed. The data were split using a hold-out strategy, allocating 60% for training, 20% for validation, and 20% for testing to ensure robust generalization.

**Preprocessing**. In brief, each model uses a distinct feature extractor: DiffAct utilizes I3D features (2048-dimensional) with the extractor proposed by Iashin et al. [37], while MS-TCN also employs I3D features but with a 1024-dimensional representation, using the extractor proposed by Carreira and Zisserman [46] and retrained by Renz et al. [21], but both require frames with size 220×220 pixels as input. Additionally, data augmentation transformations, such as rotation, zoom, and translation, were applied to improve generalization.

**Implementation Details**. The models used in this study were retrained with distinct configurations to optimize their performance. DiffAct was retrained using a batch size of 1 and a learning rate of 0.0005, while MS-TCN used a batch size of 4 and a learning rate of 0.0005. Both models were optimized using Adam. Additionally, early stopping was applied based on mF1B to prevent overfitting. The comparison between the implementation details per model is shown in Table 7.

These implementation choices reflect the specific requirements of each model—DiffAct, with its high-dimensional feature representation and generative nature, required individualized sequence processing, while MS-TCN, focused on temporal segmentation, benefited from a larger batch size and structured feature extraction.

Moreover, the models’ architectures were modified to better fit the task at hand, such that different architectural variants were tested twice and the best results on the validation set were taken as the baseline architecture for future experiments. MS-TCN was set to 4 stages, 8 blocks per stage, and 128 feature maps, while DiffAct was configured with 14 blocks and 64 feature maps for the encoder, and the decoder used 10 blocks and 64 feature maps.

Having selected the appropriate architecture for the models, we employed data augmentation techniques and multi-dataset training to improve their performance. They were applied across 10 different splits. These results were compared to those obtained without augmentation. Finally, the best-performing models for both MS-TCN and DiffAct were evaluated on the test set.

**Hardware and software**. MS-TCN was trained on Google Colaboratory using an NVIDIA Tesla T4 GPU with 12.7 GB of RAM, running in the Linux-based Google Colab environment, selected for compatibility reasons. The training setup included Python 3.11.12, PyTorch 2.6.0, and CUDA 12.4. In contrast, DiffAct was trained locally on a workstation equipped with an NVIDIA RTX 2050 GPU and 64 GB of RAM, using Python 3.9.19, PyTorch 2.0.1, and CUDA 11.

### 4.1. Architectural Modifications

DiffAct model has to be adapted to binary classification, which includes adjustments to the loss functions and output layer.

**Loss Function Adjustments.** Initially, two Binary Cross-Entropy (BCE) losses were considered:

- Classification BCE Loss—applied to distinguish between the two classes.
- Boundary Detection BCE Loss—intended to detect transition frames between classes.

However, since the task involves only two classes, boundary detection was found to be redundant and harmful to the performance, and this second BCE loss was removed. Additionally, the Mean Squared Error (MSE) loss calculation was improved. The MSE formula proposed by Liu et al. [33] was adapted to better suit the binary nature of the problem. Specifically, a probability-based transformation using the cross-entropy formula (see Equation (2)), where $y_{l}$ is the decoder’s output probability for the lth video frame, and $y_{l+1}$ is the corresponding output for the (l+1)th frame. It was introduced to determine whether predictions belonged to the same class before computing MSE. This modification significantly enhanced the performance of the model.

(2) $$\begin{array}{cc} \mathrm{cross}\;\mathrm{entropy} & =y_{l}\cdot y_{l+1}+\left(1-y_{l}\right)\cdot \left(1-y_{l+1}\right) \end{array}$$

Furthermore, the masking techniques originally proposed by DiffAct were discarded, as they neither improved nor degraded performance. In the original setting, these techniques helped the model learn logical sequences of actions, but in the context of binary classification, where no sequential structure between signs is enforced, their contribution was insignificant.

### 4.2. Ablation Study

In this section, through a series of controlled experiments, we aim to evaluate the contribution of the employed training strategies—such as baseline, data augmentation, and multi dataset-training—on boundary and sign detection. By analyzing the results under different conditions, we gain insights into the strengths and limitations of each model and better understand which factors most significantly affect performance.

#### 4.2.1. Models’ Performance with Pretrained Weights

Renz et al. [21] provide pretrained weights for various model configurations trained on different Sign Language datasets. Given the similarity among Sign Languages, particularly in terms of motion patterns, this study initially aimed to leverage these pretrained weights to improve performance on the Peruvian Sign Language datasets. The first approach involved using the model with its default weights without any modifications. However, the results were significantly poor, reaching up to 36.76% for mF1B and 32.30% for mF1S.

**Figure 8:**
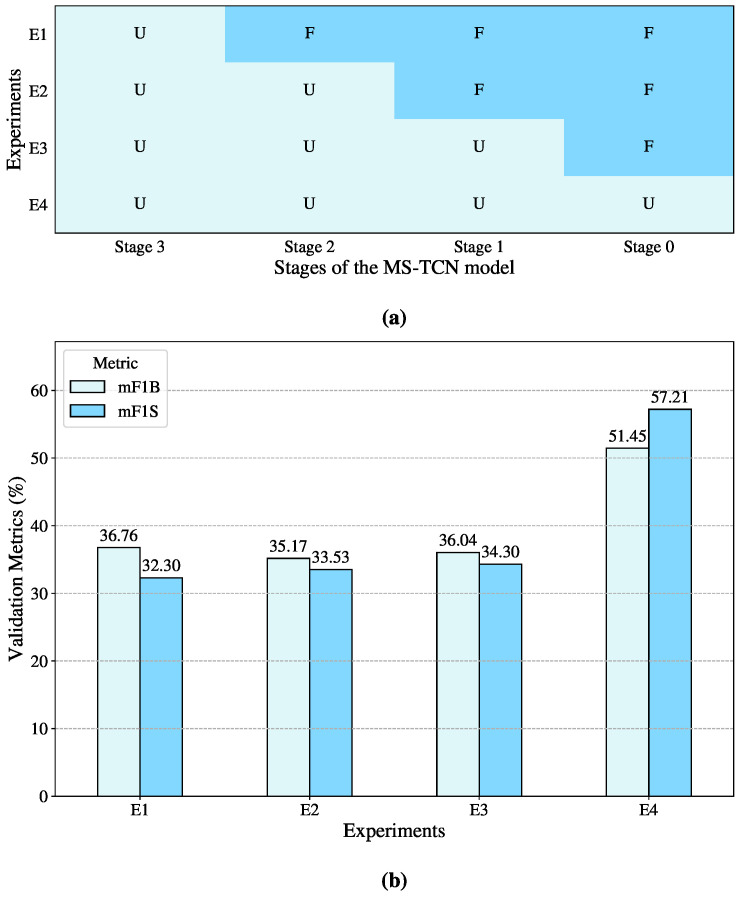
Visual representation of the progressive unfreezing of MS-TCN stages. (**a**) Illustration of the cumulative unfreezing process across the model’s four stages, where U indicates an unfrozen stage and F a frozen one. (**b**) Comparison of validation metrics (mF1B and mF1S) obtained in each experiment corresponding to different unfreezing configurations.

A gradual fine-tuning technique was used to examine the effects of pretrained representations, as shown in Figure 8a. From the last stage to the first, the four-stage MS-TCN model was gradually unfrozen, with each unfreezing step representing a different experimental environment. When the complete model was unfrozen and retrained on the Peruvian Sign Language datasets, the model performed at its peak, as seen in Figure 8b, with mF1B and mF1S values of 51.45% and 57.21%, respectively. This implies that the higher-level temporal dependencies and language-specific structures contained in the later stages of the model are not directly reusable, even though certain low-level visual features—like hand forms and motion cues—are somewhat transferable between Sign Languages.

These findings highlight an important consideration in Sign Language processing: while Sign Languages share commonalities, their grammatical structures and execution vary significantly and are heavily influenced by cultural context. Moreover, factors such as the signer’s hand movement speed, articulation style, and experience influence model performance. Some signers perform signs clearly and concisely, whereas others introduce fillers or fail to complete signs properly. These variations suggest that direct transfer learning from one Sign Language dataset to another is not always effective and must be supplemented with domain-specific fine-tuning.

On the other hand, DiffAct, being a generative model, required full retraining from scratch. Since generative models rely on the statistical properties of the training domain, applying a pretrained DiffAct model to a new dataset with different visual and motion characteristics would result in poor generalization. However, to facilitate training and leverage available knowledge, layers that matched in size with the variations in the model’s architecture were initialized with the weights from the available pretrained files. This approach aimed to accelerate convergence while ensuring the model adapted properly to the new domain.

#### 4.2.2. Baseline Performance and Tuning

Based on the performance results presented in the previous subsection, both DiffAct and MS-TCN underwent systematic architectural tuning. Multiple configurations of architectural hyperparameters were tested, and each combination was evaluated twice to ensure reliable selection. The final architectures were chosen based on the mean performance across evaluations.

As a result of this process,

- MS-TCN was set to 4 stages, with 8 blocks per stage and 128 feature maps.
- DiffAct was configured with 14 blocks and 64 feature maps for the encoder, while the decoder used 10 blocks and 64 feature maps.

These architectures served as the baseline configuration for further experiments, where we introduced additional training strategies to evaluate which approach yields improved performance over the baseline.

#### 4.2.3. Data Augmentation for Performance Improvement

The effectiveness of each augmentation technique was evaluated by training models under different augmentation configurations and comparing their impact on performance, which are summarized in Table 8.

Initially, applying all available augmentations—rotation, zoom, and translation—at the same time resulted in overfitting and no relevant improvement in the metrics. The mF1B and mF1S values were not the highest for either of the two models. The high redundancy in the transformed data likely caused the model to memorize variations instead of learning robust features, limiting its ability to generalize to unseen sequences.

For the MS-TCN model, the best metric values were achieved when applying zoom and translation transformations, resulting in an mF1B of 65.44% and an mF1S of 65.65%, along with the lowest validation loss value of 2.95.

In the case of DiffAct, the highest mF1B value of 62.68%—indicating the best performance for transition recognition—was obtained when using only zoom transformation. However, the corresponding value of mF1S, 58.59%, was significantly lower, resulting in a loss value of 1. In contrast, the combination of rotation and zoom achieved a balanced performance for both signs and transitions, with an mF1B of 60.62% and an mF1S of 60.10%, and a lower loss of 0.93.

Consequently, the combination of zoom and translation transformations will be applied to MS-TCN, while DiffAct will use the combination of rotation and zoom in subsequent experiments.

#### 4.2.4. Multi-Dataset Training

The previous experiments were conducted exclusively using the manejar_conflictos dataset. However, under this training strategy, additional videos from the ira_alegria_RE dataset were incorporated to enhance the generalization of the model. However, these samples were initially excluded due to a significant presence of the category *filler*, a phenomenon in Sign Language where movements between signs do not correspond to specific lexical signs. The excessive presence of fillers can disrupt the understanding of sign sequences, making these videos function more as noise samples rather than clear training data.

Thus, this experiment aimed not only to increase the diversity of samples but also to evaluate the effect of different interpretation styles on model performance. The manejar_conflictos dataset follows a concise signing approach, where the signer prioritizes the most relevant words to convey meaning efficiently. In contrast, the videos from ira_alegria reflect a word-by-word translation style, where the interpreter attempts to represent each spoken word with a sign. This approach sometimes results in incomplete or unnatural transitions, as certain words in Peruvian Sign Language do not have a direct equivalent or require additional context to be fully articulated.

By introducing both datasets, the experiment assessed whether the model could learn from both structured and less structured signing styles while maintaining accurate segmentation. The key challenge was to determine if the presence of filler-heavy samples negatively impacted boundary detection (mF1B) and sign recognition (mF1S) or if the increased variability in signing styles provided additional robustness to the model.

### 4.3. Selecting Optimal Models via Validation Performance of Training Strategies

The performance of both models under the three different training strategies—baseline, data augmentation, and multi-dataset—was evaluated using the Monte Carlo cross-validation technique with a fixed hold-out rate, a variation of the method originally proposed by Picard and Cook [47]. In our implementation, we generated 10 random data splits. For each split, samples were randomly selected without replacement to ensure that no sample appeared in more than one set within the same split. However, across different splits, samples could assume different roles (training, validation, or test). This evaluation setup enabled a robust estimation of model performance and facilitated the identification of the most effective training strategy.

The evaluation using validation sets and testing sets is detailed as follows in the next subsection.

#### 4.3.1. MS-TCN: Training Technique Evaluation

**Figure 9:**
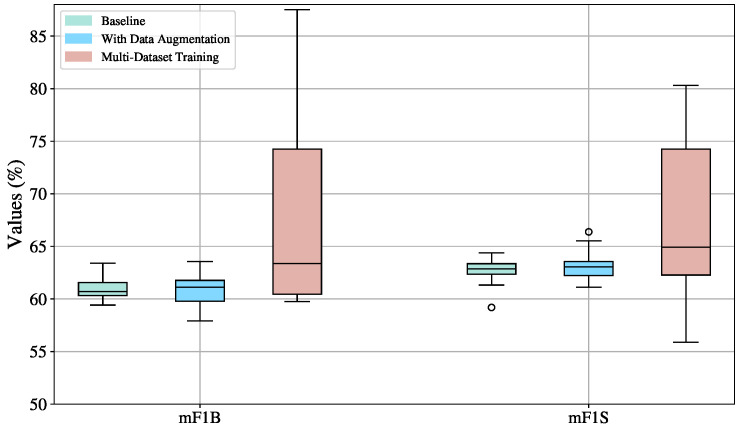
Boxplot visualization of training strategy effects (baseline, augmented, multi-Dataset) on the mF1S and mF1B metrics for the MS-TCN model, evaluated on the validation sets across ten splits.

The boxplots in Figure 9 reveal that across all settings, mF1S scores are consistently higher than mF1B, indicating that MS-TCN is more proficient at recognizing signs than detecting transitions. This suggests that the temporal structure of the model favors stable, repetitive patterns like signs over short, ambiguous ones like transitions.

Training on multiple datasets introduces substantial variability, as indicated by the highest standard deviations among all configurations: 11.18 for mF1B (%) and 12.44 for mF1S (%). Although this setup exhibits higher metric values, it also leads to instability across splits. This is visually confirmed by the wide performance ranges, from 59.76% to 87.50% for mF1B and 55.89% to 98.44% for mF1S. Additionally, the boxplots for this configuration are right-skewed, meaning that better-performing splits also exhibit greater dispersion. While some dataset combinations enhance the generalization capability of the MS-TCN model, others degrade its ability to reliably detect signs and transitions.

The configuration without data augmentation is the most stable, with the lowest standard deviations (1.20 for mF1B and 1.48 for mF1S).Yet, an outlier with an mF1S of 59.42% is located below the mean (62.61%). This could be due to an unbalanced or particularly challenging split that lacks sufficient variation in signs, emphasizing the risk of underrepresentation in small datasets, such as in this work, where the video sequences are never repeated.

Data augmentation leads to higher performance in terms of median (63.06% mF1S, 61.12% mF1B) compared to the baseline (62.87% mF1S, 60.70% mF1B), confirming its usefulness in enhancing model robustness. However, it also increases variability (standard deviation: 1.86 for mF1B and 1.67 for mF1S), suggesting that augmented data helps the model to achieve better results in some splits than in others.

Therefore, the most balanced and reliable training configuration is data augmentation, as it consistently enhances performance in both sign (mF1S) and transition detection (mF1B) compared to the baseline, while maintaining relatively low variance. This stability is crucial in practical applications, as it ensures the model performs reliably across different data splits and scenarios. In contrast, although multi-dataset training occasionally achieves high scores, the substantial variability it introduces—stemming from divergent data distributions and inconsistent generalization—renders it less dependable. Thus, data augmentation strikes an optimal balance between performance and robustness, making it the most suitable configuration for future experiments and deployments.

#### 4.3.2. DiffAct: Training Technique Evaluation

**Figure 10:**
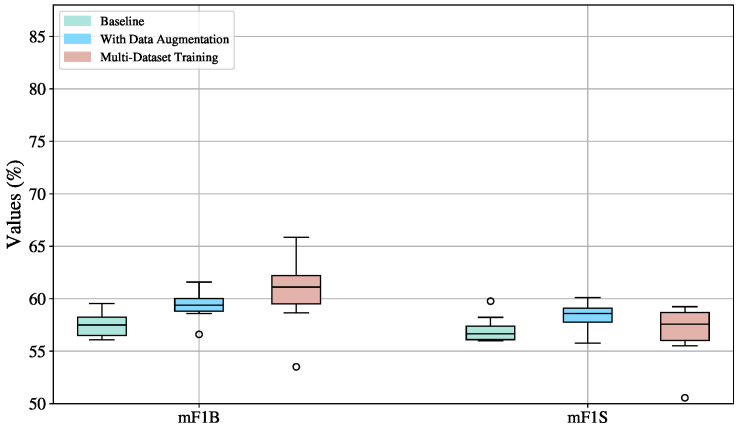
Boxplot visualization of training strategy effects (baseline, augmented, multi-dataset) on mF1S and mF1B metrics for the DiffAct model, evaluated on the validation sets across ten splits.

Figure 10 compares the performance of the Diffact model under the three training strategies: baseline, data augmentation, and multi-dataset training. In all cases, the model demonstrates better results for transition detection, as reflected in higher mF1B scores compared to mF1S, indicating the strength of the model in identifying boundaries rather than stable sign segments.

The baseline strategy exhibits the lowest variation, with standard deviations of 1.15 for mF1B and 1.21 for mF1S. However, this consistency comes at the cost of lower performance, with median scores of 60.75% for mF1B and 62.88% for mF1S. Moreover, the values are almost evenly distributed, with only one outlier in mF1S.

The use of data augmentation leads to an improvement in performance. The median for mF1B increases from 57.50% to 59.39%, and for mF1S from 56.67% to 58.58%, compared to the baseline. Standard deviations increase slightly to 1.31 for mF1B and 1.26 for mF1S.

The multi-dataset training strategy achieves the highest performance across both metrics. The median mF1B reaches 61.11%, accompanied by the highest standard deviation of 3.32. For mF1S, the median also surpasses the baseline, reaching 57.57% with a standard deviation of 2.58.

Outliers are present because a particular split comprises a sample distribution that is negatively impacted by the addition of noisy or heterogeneous data, especially when learning via data augmentation and multi-dataset training methodologies. This is probably because the distribution of the enhanced data and the original distribution of this particular split do not match. This effect is much more noticeable in the multi-dataset training situation, where the introduction of varied samples enhances the distributional shift and produces more extreme outlier values, even while data augmentation already introduces significant variability. These outliers show how poorly the model can generalize to some divides that might not be well-represented in the enhanced or merged data.

Despite its higher variability, the multi-dataset training strategy emerges as the most effective approach for training the DiffAct model. It achieves the highest median and maximum scores, particularly in transition detection (mF1B)—the most challenging class due to its brief duration and dependence on contextual cues. By exposing the model to a wider range of signing styles and scenarios, this strategy significantly enhances its generalization ability and improves its accuracy in identifying subtle transitions. While data augmentation offers a stable performance boost and the baseline ensures consistency, only multi-dataset training fully leverages the generative nature of DiffAct, enabling it to leverage noisy and diverse data for improved generalization. Therefore, despite its inherent variance, multi-dataset training is recommended as the most promising strategy for developing robust and generalizable segmentation models.

### 4.4. Testing Evaluation

**Figure 11:**
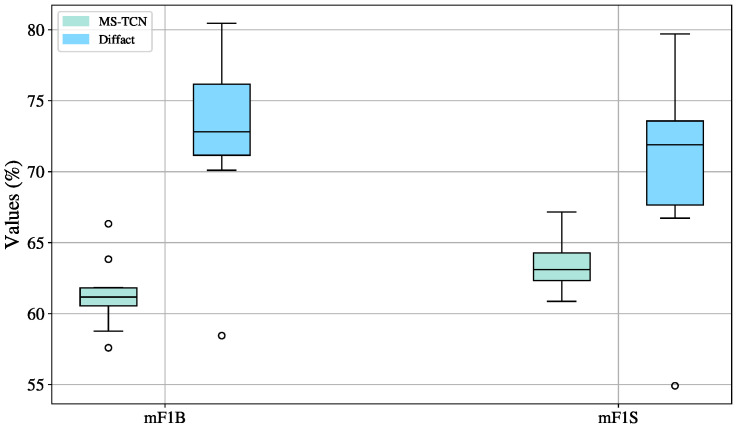
Performance of MS-TCN and the DiffAct model on the testing set across ten splits.

After selecting the best-performing configurations for MS-TCN and DiffAct based on training and validation results, we evaluated both models on the test set. As shown in Figure 11, DiffAct clearly outperforms MS-TCN in both mF1S and mF1B metrics, achieving higher medians (71.89% for mF1S and 72.68% for mF1B) and a broader upper range. In contrast, the performance of the MS-TCN model is clustered around lower medians (63.10% for mF1S and 61.17% for mF1B).

MS-TCN exhibits narrower interquartile ranges (1.94 for mF1S and 1.27 for mF1B), reflecting stable but limited performance. DiffAct, while more variable (IQRs of 5.92 for mF1S and 5.01 for mF1B), demonstrates greater potential to generalize, particularly in handling complex temporal dependencies. The presence of outliers in both models highlights occasional instability, yet the higher overall performance of DiffAct suggests that its variability is a worthwhile trade-off.

Interestingly, MS-TCN displays more outliers in mF1B, indicating inconsistent boundary detection, whereas its sign recognition (mF1S) remains stable. These findings differ from validation trends, where MS-TCN showed more favorable results, suggesting that DiffAct generalizes better to unseen data.

Despite improvements from data augmentation and multi-dataset strategies, performance variability persists, particularly in DiffAct. The small size of the dataset likely limits generalization in both models. MS-TCN appears more prone to overfitting, potentially hindering its ability to detect subtle transitions in unfamiliar sequences.

### 4.5. Qualitative Results

**Figure 12:**
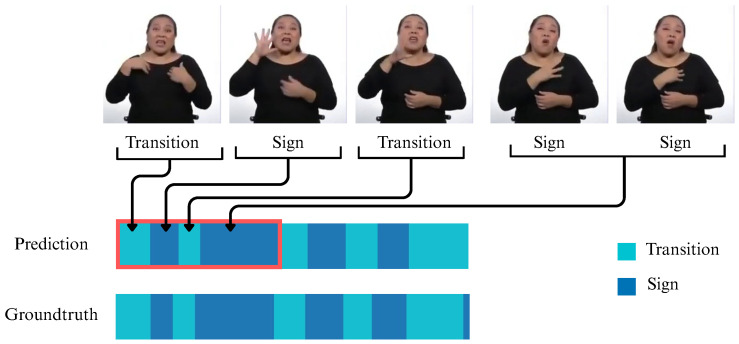
Example of model output and its correspondence to video frames.

Figure 12 presents a general example of how the results obtained in this study translate into the practical recognition of the boundaries between signs and transitions, which represent the temporal segmentation points within each video. This is demonstrated through the sample frames shown in the figure.

**Figure 13:**
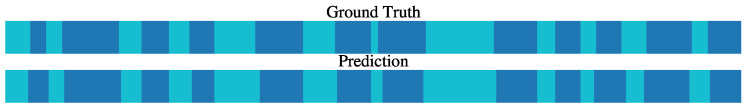
Comparison between ground truth labels (upper bar) and predicted labels (lower bar) for Video 26 from the manejar_conflictos dataset. Transitions are shown in light blue, and signs in dark blue.

**Figure 14:**
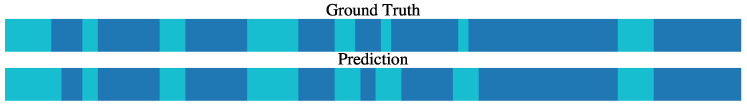
Comparison between ground truth labels (upper bar) and predicted labels (lower bar) for Video 35 from the manejar_conflictos dataset. Transitions are shown in light blue, and signs in dark blue.

**Figure 15:**
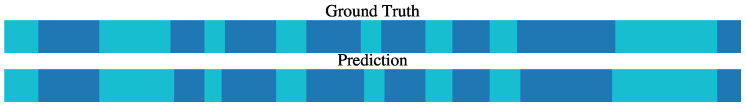
Comparison between ground truth labels (upper bar) and predicted labels (lower bar) for Video 78 from the manejar_conflictos dataset. Transitions are shown in light blue, and signs in dark blue.

**Figure 16:**
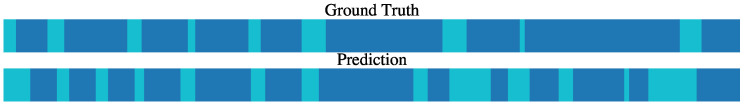
Comparison between ground truth labels (upper bar) and predicted labels (lower bar) for Video 100 from the manejar_conflictos dataset. Transitions are shown in light blue, and signs in dark blue.

Specifically, Figure 13, Figure 14, Figure 15 and Figure 16 present qualitative results for videos 26, 35, 78, and 100, from the manejar_conflictos dataset, respectively. These examples correspond to the split one, where DiffAct achieved some of its best and worst test set performance. Video 78 achieved 100% for both mF1B and mF1S, as illustrated in Figure 15, where the predicted boundaries between *signs* and *transitions*—visually distinguishable by their lighter shading—are almost perfectly aligned to the ground truth. Upon reviewing the corresponding annotations, we found that this video contains two words, out of eight, and a gestural sign that have never been encountered by the model during training.

Similarly, for video 26, we obtained 100% for mF1B and 92.71% for mF1S despite having a considerable number of signs and very brief transitions. The successful recognition of these tiny transitions may be attributed to the distinct hand configurations between consecutive signs, making the transitions more visually separable. Also, for video 35, we obtained 100% for mF1B and 92.19% for mF1S. As shown in Figure 14, the model tends to vary in a few frames when compared to the ground truth labels. However, this discrepancy is minor, as the precise start and end frames of a sign can vary slightly depending on the subjective interpretation of the annotator.

Moreover, video 100 is the sample with the lower scores for mF1B and mF1S, with 43.18% and 45.45%, accordingly. Figure 16 shows evidence of oversegmentation, where the model incorrectly predicts transitions within segments that, according to the ground truth, correspond only to continuous signs. However, upon reviewing both the annotations and the video, we found that the model correctly identified a repeated sign as two separate instances, whereas the annotator had labeled it as a single sign. Additionally, this video presents fingerspelling, but due to the low resolution and the high speed required for real-time translation, it was challenging to accurately identify the boundaries between individual letters, so the annotator considered the segments as a large sign. Despite these challenges, the model was able to detect some distinct movements that are visually similar to isolated signs.

**Figure 17:**
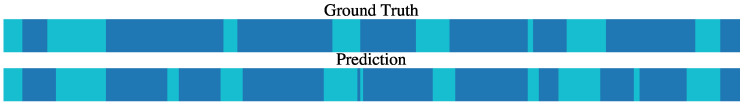
Comparison between ground truth labels (upper bar) and predicted labels (lower bar) for Video 120 from the manejar_conflictos dataset. Transitions are shown in light blue, and signs in dark blue.

**Figure 18:**
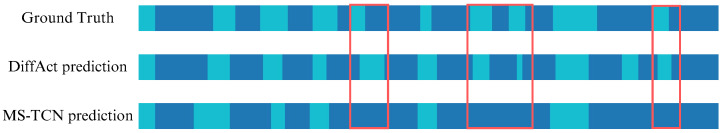
Comparison between ground truth labels (upper bar) and predicted labels for DiffAct (middle bar) and MS-TCN (lower bar) for Video 3 from the manejar_conflictos dataset. Transitions are shown in light blue, and signs in dark blue.

On the other hand, to provide a qualitative comparison between DiffAct and MS-TCN, we selected split 8—the split in which MS-TCN achieved its highest overall performance—and analyzed both its best and worst cases. As shown in Figure 17, MS-TCN performs well in sequences with fewer class transitions and longer, more stable segments. In contrast, Figure 18 presents a challenging case involving short transitions and frequent class changes, where MS-TCN struggles, achieving 53.12% for mF1B and 35.94% for mF1S, it fails to detect several transitions, highlighted by red rectangles, where hand configurations between consecutive signs change only slightly, making boundaries difficult to identify. In the same scenario, DiffAct demonstrates superior robustness to rapid temporal variations, with significantly higher scores of 78.57% for mF1B and 53.57% for mF1S.

This improvement can be attributed to DiffAct’s diffusion-based architecture, which models temporal dynamics as a denoising process across the entire sequence. This global modeling allows it to better capture subtle transitions and adapt to rapid changes in class boundaries. Unlike MS-TCN, which relies on stage-wise refinement that may overly smooth frequent transitions, DiffAct’s iterative refinement of latent representations enables more precise boundary localization in complex temporal patterns.

## 5. Conclusions and Future Work

This study evaluated the effectiveness of two temporal segmentation models—MS-TCN [21] and DiffAct [33]—in the context of Peruvian Sign Language video segmentation. Through experiments involving fine-tuning, data augmentation, and multi-dataset training, we found that diffusion-based models like DiffAct, despite their higher variability, offer strong potential for handling complex temporal patterns in sign segmentation tasks.

As part of this work, we introduced three novel Peruvian Sign Language datasets: manejar_conflictos, ira_alegria_RE, and PUCP-305_RE, based on publicly available videos and annotated in collaboration with a certified Peruvian Sign Language interpreter. The annotation process was resource-intensive—requiring roughly one hour per minute of video—due to the need for linguistic and cultural precision. In addition to the core classes of *sign* and *transition*, the data revealed other important categories, including *fillers*, *NN* (incomplete signs), and *gestural signs*.

We publicly share our datasets and code to support reproducibility and encourage integration into active learning pipelines. Our models can serve as pre-annotation tools, potentially assisting human annotators and advancing sentence-level translation in Continuous Sign Language Recognition tasks.

For future work, we propose exploring hybrid architectures that combine the temporal stability of MS-TCN with the generative strengths of DiffAct. Transformer-based models also present a promising direction due to their capacity to model long-range dependencies. Further improvements could include designing data augmentation strategies that maintain temporal coherence, incorporating linguistic features such as facial expressions and handshape transitions and refining loss functions for better boundary detection.

Domain adaptation techniques—such as adversarial learning and distribution alignment—could help adapt pretrained models to new datasets and improve generalization across different Sign Languages. Moreover, evaluating the practical impact of these models on annotation efficiency by involving interpreters in user studies will be essential for assessing real-world benefits.

Finally, expanding national datasets is crucial, as Sign Languages vary widely across regions. We plan to extend our datasets with a greater number of signers, more diverse sentence structures, and better representation of minority classes. This will support signer-independent evaluation and open avenues for multiclass classification, ultimately enhancing the robustness and applicability of segmentation models in real-world scenarios.

## Figures and Tables

**Table 1:** Comparison of related work on temporal Sign Language segmentation.

Approach	Work	Main Method	Dataset
Sign Language temporal segmentation	Mocialov et al. [23]	Calculation of hand centroids acceleration and motion classification	NGT corpus (Sign Language of the Netherlands)
	Choudhury et al. [24]	Calculation of hand centroids acceleration and motion classification	Homemade videos
	Nayan et al. [25]	Optical flow for velocity estimation	Indian Sign Language (fingerspelling)
	Farag and Brock [26]	3D joint modeling + balanced Random Forest	DJLSC, CMU Mocap
	Renz et al. [21]	Hierarchical model	BSL-Corpus, PHOENIX14
	Pérez et al. [22]	Retrained transformer with attention mechanism	PHOENIX14
Action temporal segmentation	Bahrami et al. [32]	Combines local and long-term attention mechanisms	Assembly101, 50Salads and Breakfast
	Liu et al. [33]	Diffusion model + dilated convolutions	GTEA, 50Salads and Breakfast
	Yang et al. [27]	3D Convolutional Network	THUMOS14 and UCF101
Continuous Sign Language Recognition	Feng et al. [8]	Dynamic module + Bi-LSTM + contrastive loss	PHOENIX14-T, PHOENIX14 and CSL-Daily
	Huang et al. [35]	Hierarchical Attention Network with Latent Space	CSL, PHOENIX14

**Table 2:** Comparison between Peruvian datasets.

Dataset	# Videos	# Unique Sentences	Annotated Time (minutes)	# Signers	Level of Annotation
AEC PUCP	2	270	18.05	2	Word and sentence
PUCP-305	272	272	≈18.13	5	Word and sentence
LSP-10	600	10	0	25	None

**Table 3:** Summary of datasets used in this study.

Datasets	# Sentences	Annotated Time (Minutes)	Mean Sentence Duration (Seconds)	# Signers
manejar_conflictos	171	21.17	8.36	1
ira_alegria_RE	201	20.97	9.82	1
PUCP-305_RE	174	15.50	5.80	5

**Table 4:** Summary of the notation used during the manual annotation process.

Categories	Description
Sign	A meaningful gesture formed by a combination of manual and non-manual features
Transition or movement epenthesis (ME)	A transitional movement that occurs between two signs
Rest	Resting position
Fillers	Involuntary and/or repetitive hand movements that lack meaning
NN	Signs that are performed incorrectly or incompletely
Gestural signs	Movements that accompany a sentence to express non-linguistic aspects

**Table 5:** Number of video frames per category for every dataset considered in this study.

Dataset	Rest	Fillers	NN	Gestural Signs	Signs	Transitions
manejar_conflictos	160	47	20	41	32,261	14,978
ira_alegria_RE	45	366	51	63	35,740	13,687
PUCP-305_RE	366	0	14	19	11,296	18,553

**Table 6:** Data augmentation methods and the range of variation.

Augmentation Method	Range
Rotation	−10° to 10°
Zoom	0.8% to 1.2%
Translation in X and Y axis	−15 to 15

**Table 7:** Implementation details of the retrained models.

Parameter	DiffAct	MS-TCN
Feature Extractor	I3D (2048D)	I3D (1024D)
Learning Rate	0.0005	0.0005
Batch Size	1	4
Optimizer	Adam	Adam
Early Stopping	Yes (Metric: mF1B)	Yes (Metric: mF1B)
Function Loss	MSE and BCE	MSE and BCE

**Table 8:** Performance results using different combinations of data augmentation techniques for DiffAct and MS-TCN models. Best results are in bold.

Augmentation Techniques			MS-TCN		DiffAct	
Rotation	Zoom	Translation	mF1B	mF1S	mF1B	mF1S
✓			62.47	61.35	60.04	59.53
	✓		63.92	63.65	62.68	58.59
		✓	64.16	63.57	60.85	57.41
✓	✓		63.18	62.84	**60.62**	**60.10**
	✓	✓	**65.44**	**65.65**	60.56	60.17
✓		✓	62.55	62.82	60.52	58.01
✓	✓	✓	62.55	62.09	60.89	59.11

mF1B: mean F1 Boundary; mF1S: mean F1 Sign.

## Data Availability

The data supporting the findings of this study will be made available upon reasonable request to the corresponding author. **Code Availability**: The code used for this study can be accessed at the following link: https://github.com/Roml68/Temporal-segmentation-for-Peruvian-Sign-language.git (accessed on 6 February 2025).
